# 3D in vitro models of skeletal muscle: myopshere, myobundle and bioprinted muscle construct

**DOI:** 10.1186/s13567-021-00942-w

**Published:** 2021-05-19

**Authors:** Frederic Dessauge, Cindy Schleder, Marie-Hélène Perruchot, Karl Rouger

**Affiliations:** 1 PEGASE, INRAE, Institut Agro, 35590 Saint-Gilles, France; 2 INRAE, 44307 Nantes, Oniris, PAnTher France

**Keywords:** Skeletal muscle, Satellite cell, Myoblast, Muscle-derived cell, Myosphere, Myobundle, 3D bioprinting, Scaffold, Extracellular matrix, Hydrogel, Bioink

## Abstract

Typical two-dimensional (2D) culture models of skeletal muscle-derived cells cannot fully recapitulate the organization and function of living muscle tissues, restricting their usefulness in in-depth physiological studies. The development of functional 3D culture models offers a major opportunity to mimic the living tissues and to model muscle diseases. In this respect, this new type of in vitro model significantly increases our understanding of the involvement of the different cell types present in the formation of skeletal muscle and their interactions, as well as the modalities of response of a pathological muscle to new therapies. This second point could lead to the identification of effective treatments. Here, we report the significant progresses that have been made the last years to engineer muscle tissue-like structures, providing useful tools to investigate the behavior of resident cells. Specifically, we interest in the development of myopshere- and myobundle-based systems as well as the bioprinting constructs. The electrical/mechanical stimulation protocols and the co-culture systems developed to improve tissue maturation process and functionalities are presented. The formation of these biomimetic engineered muscle tissues represents a new platform to study skeletal muscle function and spatial organization in large number of physiological and pathological contexts.

## Structural organization and repair process of skeletal muscle tissue

Skeletal muscle is the most abundant tissue, representing 35–45% of the total body mass [1]. It functions to generate force that enables locomotion, posture, swallowing, and breathing. It is composed of a mixture of terminally differentiated fibers that represent the basic contractile units grouped into bundles. These multinucleated muscle fibers are highly oriented with one another and correspond to a single long cylinder. They are each contacted by a single motor neuron (MN) and express different myosin heavy chain (MHC) isoforms and metabolic enzymes that are responsible for their own contractile properties [2]. They are individually surrounded by a connective tissue layer and by an important vascular network that allows the supply of nutrients. Then, the functional characteristics of skeletal muscle are directly linked to the organization of this complex framework of fibers, MNs, extracellular connective tissue matrix, and blood vessels [3]. Post-natal skeletal muscle is a terminally differentiated tissue with very little turnover of nuclei, less than 1–2% of them being replaced per week [4]. Moreover, it was characterized by a remarkable ability to ensure a rapid and extensive repair in response to injury, preventing the loss of muscle mass. This repair, which corresponds to a finely orchestrated regenerative process, requires the activation of various cellular responses necessary for the formation of a well innervated, fully vascularized, and contractile muscle apparatus. Among them, activation of satellite cells (SCs), which are undifferentiated myogenic progenitors residing between the basal lamina and sarcolemma [5], is a key element in this process [6, 7]. Following muscle fiber damage, they activate and proliferate before differentiating and fusing with existing fibers in order to repair the injured area [8, 9]. Since their first observation in the *Tibialis anterior* muscle of the Frog [10], several markers have allowed identification of SCs in many species including Human [11, 12], Mouse [13], Rat [5], Monkey [14], Pig [15], Cattle [16], Chick [17, 18], Salamander [19], and Zebrafish [20] and Rainbow trout [21] (Table 1).

**Table 1:** **List of studies describing experimental protocols to isolate satellite cells and to expand primary myoblasts in different specie﻿s**.

Specie	Satellite cell	Myoblast
Frog	[10]	[22]
Rat	[5]	[23]
Mouse	[13]	[24]
Human	[11, 12]	[25]
Monkey	[14]	[26]
Pig	[15]	[27]
Cattle	[16]	[28, 29]
Chicken	[17, 18]	[30]
Salamander	[19]	[31]
Zebrafish	[20]	[32]
Rainbow trout	[21]	[33]

## Limitations of 2D cultures and interest of bioengineered skeletal muscle tissue

Conventional two-dimensional (2D) cell culture relies on adherence to a flat surface, typically a Petri dish of glass or polystyrene, to provide mechanical support for the cells. Cell growth in 2D monolayers allows for access to a similar amount of nutrients and growth factors present in the medium, which results in homogenous growth and proliferation, in highly controlled condition. In 2D systems, primary cultures of myoblasts (i.e., myogenic precursors corresponding to natural descendants of SCs) have been developed in Frog [22], Rat [23], Mice [24] and Humans [25] as well as in a wide range of species, including Monkey [26], Pigs [27], Cattle [28, 29], Chicken [30] and Fish [31–33] (Table 1). If such cultures are well known and under control, in return they display several critical limitations, among which a non-sustainability over the long-time, a lack of native muscle architecture, and a difficulty to produce spontaneous contractions [34, 35]. Then, they do not fully recapitulate the structural organization and function of adult muscle that are yet essential for the muscle contraction and functionality [36, 37]. This limits the use of 2D culture systems in biological studies as well as in in vivo replacement of pathological or damaged tissues [38, 39]. In addition, cell culture with current conventional planar in vitro systems presents significant limitations related to their low surface to volume ratio, the lack of pH, gas and metabolite concentration control and is therefore not scalable [40]. Hence, development of 3D in vitro systems is motivated by the need to alleviate ethical considerations demanding a reduction in the use of animals and improve outcomes in human patients by identification of novel therapeutic thanks to drug screening and cell-based assays [41, 42].

## Development of 3D model of primary muscle-derived cell culture: myosphere

Over the last decades, the efficacy of cell-based therapeutic strategies for muscle disorders as well as the production of generic knowledge on the biology of muscle stem cells have been hampered by recurrent difficulties in isolating and propagating tissue-resident stem cells in their native state, which are yet essential for relevant treatment and banking of biological material. Indeed, in conventional 2D culture conditions, expanded muscle-derived cells (MDCs) rapidly mature and lose their ability to engraft [43]. Also, the mechanisms balancing quiescence, self-renewal, and differentiation of SCs are difficult to analyze in vitro because their staminality is lost when they are removed from the niche and is not adequately reproduced in the 2D culture models currently available. For these reasons, culturing method in suspension was developed with the objective that the 3D cell–cell interactions would provide a niche-like environment to help maintain cells in a more primitive state [44].

**Figure 1:**
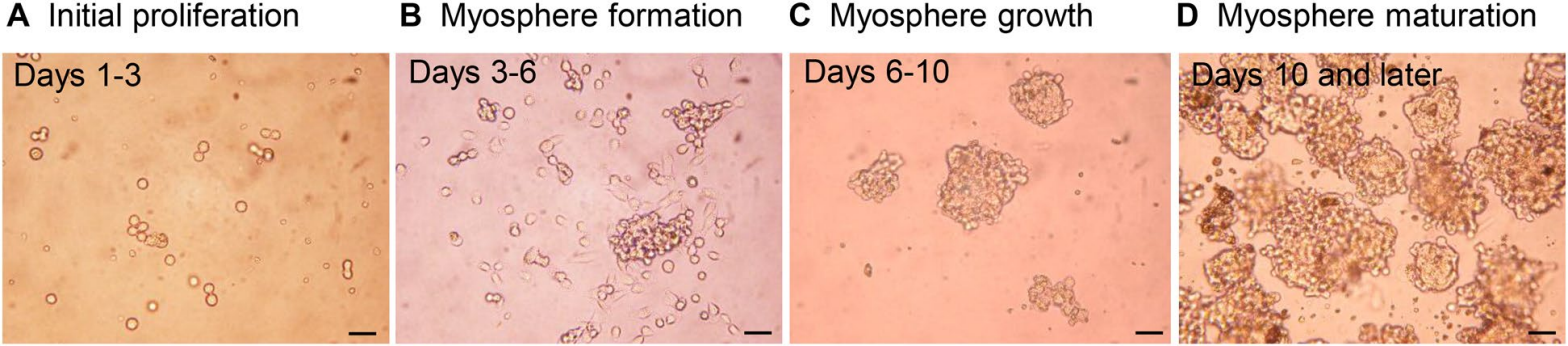
**Phase contrast microscopy of classical myosphere cultures.** **A** When cultured in suspension, progenitor/stem cells initially correspond to very few small and refringent rounded cells. Within the first days (days 1–3) some of them could appear in clusters of two to four cells. **B** Then (days 3–6), they began to grow according to atypical modalities as they aggregated into microspheroid colonies composed of a few dozen of joined cells that occasionally could also appear superposed. **C** In the following days (days 6–10), these floating myospheres pursued to grow in size and number, reaching several hundreds of cells. **D** After 10 days, the myospheres appeared as spheroid complexes that are very dense in cells and voluminous. They can be maintained by serial passages as suspended myospheres for at least several weeks without losing their proliferation ability. Scale bars: 25 µm. Related to the work done on in vitro characterization of canine MuStem cells [26].

Myosphere corresponds to 3D structure generated from freshly isolated MDCs and that gradually appeared from 3 days after initial seeding of mononucleated cell suspension as recapitulated in Figure 1 [45–49]. In the first days after muscle tissue isolation resulting from mechanical and enzymatic dissociation, small round cells, mainly individual, were seen floating among the debris. Over the next few days, the single or pair cells become small clusters of few cells, then larger clusters of dozens of cells and later forming rounded sphere-like structures. These myospheres were initially 25–50 μm in size and continued to grow over time reaching sizes as large as 200–400 μm. Then, myospheres are propagated in vitro as free-floating clusters of rounded cells exhibiting different diameters. They can be passaged every 20–30 days by trapping spheres that are > 100 μm, dissociating them with dispase/collagenase and then plating the cells at a density of 1–10 × 10^5^ cells/mL. The newly replated myosphere cells will form new free-floating spheres over a period of several days. Characterization of myosphere-based cultures revealed that they contain myogenic cells expressing α7-integrin, Pax7, Myf5 and MyoD but also non-myogenic cells defined as α7-integrin^−^, platelet-derived growth factor receptor alpha^+^ and stem cell antigen-1 (Sca-1)^+^. It was suggested that this second cell type corresponds to an interstitial cell population [47, 50]. Interestingly, it was determined that cell derived from myospheres behaved similar to primary myoblasts in that they mostly express Pax7, Myf5 and MyoD as well as form multinucleated myotubes when cultured adherently [51]. However, some differences in terms of proliferation rate, differentiation capacity and phenotype were presented between the myospheres generated by several groups, depending on isolation procedure and culture media used [45, 46, 50, 51]. Notably, the addition of leukemia inhibitory factor to the growth medium of the myospheres enhances proliferation and dramatically increases the proportion of cells expressing Sca-1 [45].

Interestingly, it was established that some of MDCs in myospheres are able to maintain a pre-myogenic state in culture over time [47]. As an example, myospheres generated from human neck skeletal muscle cells can be cultured and expanded for 20 weeks or 18 passages even when obtained at an average donor age of 63 years, demonstrating a sustained self-renewal capacity [49]. Furthermore, culturing myospheres can be isolated from the muscles of young and old mice and can be obtained over extended periods of time (3–4 months) [51]. It was evoked that the 3D adhesive cell–cell interactions involved in maintaining the sphere-like myosphere structures are also involved in maintaining their longevity in vitro [50].

Thanks to their 3D structure, myospheres allow exhibiting cell–cell interactions and spatial organization between cell types closer to that observed in tissues compared to classical 2D culture systems. In contrast to 2D MDC-derived primary cultures in which progenitors are rapidly lost due to their low division time compared to the other resident cell types, a main advantage attributed to the 3D formation of myospheres resides in the maintaining of cell proportion between progeny [49]. Another advantage is that the initial culture procedure is simple and relatively gentle with little manipulation of the freshly extracted MDCs. This contrasts with many SC isolation procedures that incorporate the use of flow cytometry, which is known to activate the SCs and thus alters their gene expression profile [52, 53]. As an example, the expression of notch1, which plays a pivotal role in determining SC behavior, was shown modified in SCs [54]. Regarding the contribution of myospheres to muscle regeneration, it was shown that myosphere-derived cells participate in forming new muscle fibers and generating SCs when injected into damaged muscles [24, 46]. Also, the authors mentioned that they exhibit a grafting capacity superior to that of myoblasts. Consistent with these findings, Westerman et al. demonstrated that injection of GFP^+^ myosphere-derived cells into dystrophic mice muscle generate the formation of GFP expressing muscle fibers 2 weeks later [47]. Interestingly, these results pointed out that myospheres-derived cells have the capacity to regenerate injured muscle in vivo in addition to their ability to retain stem cell properties during in vitro expansion, supporting that myosphere-derived cells may be highly useful in regenerative medicine for skeletal muscle diseases.

## Development of 3D skeletal muscle models: myobundle

Since pioneering works leading to myotube formation in a collagen gel [55] and 3D scaffold construction [56], the field of skeletal muscle tissue engineering has greatly developed. Engineering approach is based on the exploitation of two main components corresponding to a biomaterial that ensures suitable tissue scaffolding and the tissue-resident cell types. Concerning bioscaffold materials, they must be inert, resorbable by biodegradation processes, modifiable, and exhibit minimal cytotoxicity [57, 58]. Also, optimal biomaterials should contain a high surface area for cell adhesion, support mechanical integrity of the tissue, minimize diffusion distances, and degrade once there is no need for structural support [59, 60]. Bioscaffolds harvested from naturally occurring sources (i.e., extracellular matrix [ECM]) or created by artificial means using synthetic materials such as poly-L-lactic, polylactic-glycolic, and polyurethane [61–63]. The main advantage of the synthetic scaffolds over naturally derived materials resides in the fact that they can be precisely characterized and fabricated with great control over physical and chemical properties. Hybrid devices have also been attempted as scaffolds for muscle tissue generation [64, 65].

The three main types of scaffolds used for the production of myofiber bundles are successively presented below before the presentation of bioengineering protocols developed to increase the maturation and functionality of engineered skeletal muscle.

### Native ECM proteins-based scaffold

Interactions between cells and ECM, which mainly consists of collagen I-IV, fibrinogen, laminin and glycoaminoglycans as well as an assortment of growth factors, contribute to critical cellular processes during normal muscle growth and regeneration [66, 67]. Moreover, naturally derived hydrogels (collagen, fibrin, Matrigel) were shown to well support high density and 3D spreading of muscle cells [68], unidirectional cell alignment through the application of geometric constraints [69] and macroscopic tissue contractions [70, 71]. Then, native skeletal muscle-derived ECM proteins have been largely used in the past as a component of scaffold material [63, 72]. As an example, in a 3D setting, the Bursac’s lab has tested various matrix protein type (collagen I/fibrinogen/Matrigel) with different concentrations in hydrogel-based neonatal rat skeletal muscle bundles to assess their respective impact on tissue structure, generating of contractile force and intracellular Ca^2+^ handling [73]. Engineered muscle tissue has been prepared based on a hydrogel molding technique initially developed by Rhim et al. [74]. Tissue molds were fabricated by longitudinally splitting a 25 mm long section of 4.7 mm diameter silicone tubing and sealing both ends with a small piece of polydimethylsiloxane (PDMS). Molds were sterilized with ethanol, submerged in 0.2% (w/v) pluronic F-127 solution to prevent gel adhesion, dried with nitrogen, and placed in a standard 6-well tissue culture dish. Velcro tabs, which served as gel attachment sites and allowed generation of passive longitudinal tension in the bundles, were sterilized with ethanol and secured at both ends of the mold with stainless steel pins. After initiation of the fibrin gel polymerization as a result of thrombin addition, mixed cells/gel solution was injected into a silicone tissue mold and incubated at 37 °C until gelation. The polymerized cell/gel bundles were then maintained in growth medium for 5 days, then switched to low serum differentiation medium. After two weeks of culture, the muscle bundles consisted of highly aligned and cross-striated muscle fibers and exhibited standard force–length and force-frequency relationships achieving tetanus at 40 Hz. The use of fibrin yielded higher isometric tetanus amplitude as compared to those measured in collagen I-based bundles. Adding Matrigel to collagen hydrogels improved engineered muscle structure while high collagen content has adverse effects on muscle maturation. Also, higher fibrin and Matrigel concentrations synergistically yielded further increase in active force generation. Unlike collagen that has been initially used by most of the laboratories, fibrin was presented as to be the best choice as a matrix for tissue engineering of skeletal muscle due to its ability to be extensively remodeled and degraded [75, 76] as well as its stiffness comparable to that of native muscle [77, 78]. It promotes angiogenesis and neurite extension, which is critical for the formation of a fully functional engineered muscle for in vivo applications [53, 79].

### Fibrin hydrogel

In 2015, the Bursac’s lab generated the first biomimetic human skeletal muscle culture system (“myobundle”), using cells casted within a fibrin/Matrigel matrix and anchored to nylon frames [80]. The myobundles began to spontaneously twitch after 3–5 days culture and contained densely packed and aligned myofibers surrounded at the periphery of fibroblasts after 2-week culture. Mature structure of the myofibers was evident by the expression of MHC, cross-striations, and multiple myogenin + nuclei. Of functional importance, acetylcholine receptors (AChR) were present at the myofiber surface. A fraction of cells continued to express the SC marker Pax7, suggesting regenerative capacity as previously described in a rat culture model [81]. In the same way, the Gilbert’s lab demonstrated an enhancement of fiber maturation and AChR clustering following myogenic differentiation in 3D culture [82]. The authors established primary myogenic progenitors and CD56^−^ fibroblast-like cells from human biopsy tissues and seeded them at defined ratios either within fibrin/Geltrex hydrogel (3D) or into 12-well tissue culture plastic dishes coated with Geltrex (2D) or fibrinogen/Geltrex blend. Muscle cell laden hydrogels were formed within a PDMS channel and anchored at each end of the channel to the nylon hooks of Velcro fabric, which act as artificial tendons and establish uniaxial tension during 3D tissue remodeling and differentiation. While 2D muscle fiber cultures were regionally aligned but globally disorganized, a uniform alignment of striated muscle fibers was determined along the tension axis in the 3D tissues. In contrast to the muscle fibers established in 2D cultures, those derived in 3D culture progressively increased in diameter over 3 weeks in culture while maintaining fiber alignment and assembled contractile apparatus. In addition, an upregulated expression of the fast and slow adult isoforms of MHC was noted, suggesting a gradual sarcomere structural maturation. 3D human muscle tissues were capable of generating active force in as early as 10 days of differentiation as evidenced by spontaneous twitches, which were not observed in 2D cultures. ACh stimulation produced an immediate tetanus response in 3D tissues suggesting an abundance of active AChRs, while the response of 2D muscle fiber cultures at this time point was significantly less and inevitably resulted in muscle fiber damage.

Maffioletti et al. generated 3D artificial human skeletal muscle by embedding in fibrin hydrogels and differentiating pluripotent cells-derived myogenic cells (from healthy and dystrophic patients) [80], using an adaptation of a cardiac tissue engineering platform to direct orientation of cells along the force axis [83]. Over 10 days, cells remodeled the matrix and generated a 7–8 mm long strip of tissue containing structures that resemble skeletal muscle fibers. Similarly, under optimized 3D culture conditions, myogenic progenitors derived from multiple human pluripotent stem cell lines were shown to reproducibly form functional skeletal muscle tissue bundles containing aligned multi-nucleated myotubes that exhibit positive force-frequency relationship and robust calcium transients after electrical or ACh stimulation. Bundles exhibited increased structural and molecular maturation, hypertrophy, and force generation for 4 weeks in vitro. Following implantation into hindlimb muscle of immunocompromised mice, bundles demonstrated an ability to survive, progressively vascularize, and maintain functionality [84]. In vitro formation of such bundles represents an interesting microphysiological platform for human muscle disease modeling and drug development. In agreement with these findings, other studies confirmed that the use of 3D environment is associated with longer culture times [80, 84, 85], increased myotube size [86], increased protein content [55], and improved maturation of MHC gene expression compared to 2D cultures [84].

### Methacrylated gelatin hydrogel

Hosseini et al. determined that a micropatterned methacrylated gelatin (GelMa) hydrogel was a suitable substrate to align C2C12 myoblasts and to generate functional skeletal muscle tissue [87]. GelMA solution was prepared from the dissolution of the gelatin type A in methacrylic anhydride. To generate micromolded GelMA hydrogels, it was poured into polystyrene Petri dish after that a PDMS stamp corresponding to a photoresist mold of a 100-μm grooves/50-μm ridges pattern was applied. The pattern, which was made on a silicon wafer by a conventional photolithographic technique [88, 89], measured 50-μm in height and covered a surface of 1 cm^2^. The GelMA pattern was then photocrosslinked under UV and the PDMS stamp was gently removed. C2C12 myoblasts that were cultured on the micropatterned GelMA hydrogels were significantly more aligned on day 3 of culture than the control cells cultured on non-patterned hydrogels. GelMA hydrogels were presented as high-performance materials for tissue engineering developments by allowing cultured cells to migrate, proliferate, and contact with each other. Also, their mechanical properties are tunable and their high diffusion capacity through their pores is advantageous for cell nutrition and waste removal.

### Methods to increase engineered skeletal muscle maturation and function

Electrical and mechanical stimulations. Although myobundles recapitulated many of the morphological and functional features of native skeletal muscle, their size and isometric contractile properties were, however, inferior to those of native adult muscle, revealing an incomplete muscle maturation [90, 91]. Under long-time electrical stimulation, primary culture of myoblasts developed on fibrin or polylactic acid micropatterns displayed enhanced myotube formation and a greater rate of differentiation [92, 93]. In addition, increased myobundle maturation and force were established in rodents [94, 95] and humans [96] 3D-engineered muscle tissues. For that, electrical stimulation was performed between weeks 1 and 2 of myobundle differentiation using 1-h stimulation bouts separated by 7-h rests. Specifically, myobundles were continuously electrically stimulated at 1 Hz or with a 0.5 s, 10 Hz pulse train every 5 s, thus delivering the same total amount of stimulation pulses. Similarly, use of cyclic loading or stretch protocols revealed that mechanical stimulation also induces muscle hypertrophy and protein content [97] as well as increases engineered muscle differentiation and function [98].

Co-culture systems. To improve the formation of mature muscle tissues with higher functionalities, different co-culture systems have been developed in bioengineering protocols. C2C12 myoblasts cultured in micropatterned GelMa hydrogels with PC12 neural cells showed improved differentiation with enhanced myotube formation, alignment and length [99]. At the molecular level, an up-regulation of markers specific to muscle differentiation, maturation and neuromuscular junctions (NMJ) were also noted. Improved vascular organization was established in tri-culture composed of myoblasts, fibroblasts and endothelial cells [100]. Other studies, using a co-culture of C2C12 and endothelial cells, have shown the development of prevascularized tissue with the formation of a capillary network, allowing a quick functional anastomosis when transplanted [101]. The NMJ is a highly organized synapse formed between a MN axon and a muscle fiber, which is responsible for the transmission of efferent signals from projecting MNs to muscle fibers in order to actuate fiber contraction. In 2019, the Gilbert’s lab reported a method integrating architectural cues with co-culture techniques to create an environment conducive to the de novo formation of the adult human NMJ as early as 2 weeks [82]. MN clusters were mixed with myogenic progenitors in the hydrogel mix, and seeded together into the PDMS channels. After 10 days in differentiating media, co-cultures self-organized such that myogenic progenitors fused to form multinucleated, aligned and striated muscle fibers and the MN clusters were positioned at the periphery of muscle bundles. Importantly, the MNs were capable of regrowing neurites that were found in contact with AChR clusters on muscle fibers. Western blot analysis confirmed expression of MuSK and rapsyn proteins, two decisive synaptic proteins for mediating agrin-induced synaptogenesis. More and larger AChR clusters in 3D co-cultures were observed as compared to 3D muscle alone cultures, particularly at sites where MN neurites contacted muscle fibers. Overall, in side-by-side studies of muscle fibers cultured in 2D, the authors showed that the 3D culture system enables long-term maintenance of maturing muscle fibers in culture.

## 3D bioprinting, another strategy to generate functional skeletal muscle tissue constructs

Over the last years, bioprinting techniques have emerged to produce 3D platforms with or without cells. Their main advantages lie on their reproducibility, suitability, accuracy but also the ability to bioengineer various functional tissue constructs with complex geometry by building up cell-laden hydrogels in a layer-by-layer fashion [102–104]. Several microfabrication methods have been developed to generate biomimetic scaffolds that can provide the same topological cues as muscle tissues at the micron scale, such as electrospinning, micromolding, photolithography and soft lithography methods [105]. Briefly, electrospinning is a method widely applied in the synthesis of tissue engineering scaffolds as it can both control the size, morphology of nanofibers by adjusting the technical parameters and use almost every soluble polymer and additive [106–108]. Using a wet electrospinning system, a core–shell 3D composite scaffold that contained an aligned nanofiber yarn core was designed [109]. In this context, C2C12 myoblasts showed well alignment and correctly developed into a 3D elongated myotube formation. The micromolding approach displays the advantages of short processing time and easy-to-use procedures with the possibility to employ either elastomers such PDMS or other materials as templates for the creation of tissue constructs [110]. Sinusoidally waved/patterned PDMS substrates were shown to allow C2C12 myoblasts forming well-aligned myotubes after 6 days. Same results have been obtained on alginate microfibers with patterned ECM protein adhesive sites (fibronectin) generated using micromolding and a microcontact printing system [111]. Photolithography is a process to transfer geometric features of a mask to a substrate by using a photoresist and a light source [112–114]. When photoresists are exposed to UV irradiation with or without optical mask, the patterns are transferred from optical mask to materials, allowing the production of scaffolds for tissue engineering [115, 116]. Soft lithography relies on the use of a soft elastomeric stamp with a micropatterning whom multiple copies can be prepared by photolithographic process, making it more convenient and cheaper than standard photolithography [117, 118].

In parallel, 3D bioprinting methods have been widely studied the last decade in order to stack living muscle cells and biomaterial layer-by-layer that helps the cell growth and cell signals through modulation of the cell–cell interaction and cell–matrix interaction [119]. These methods represent a powerful manufacturing technology for tissue engineering because they can easily fabricate bulk and complex tissue, mimicking the structure and endowing cells with a biomimetic 3D microenvironment. The conventional methods can be classified into three main groups corresponding to inkjet, microextrusion, and laser-assisted methods [120, 121]. Concerning the inkjet method, the most commonly used approaches to eject bioink onto a substrate are the thermal- and piezoelectric-nozzle approaches. The resolution of the printed constructs may however be limited due to the nozzle-injection style and material characteristics [122]. With the microextrusion method, the techniques typically used for the dispersion of the biomaterials onto a substrate are pneumatic-, piston- and screw-dispensers [123]. Construction of large free-form tissue structures appeared to be quite difficult due to inadequate mechanical stability and printability [124]. The laser-assisted method has the advantage to be nozzle-free, which can avoid clogging observed with the previous methods, but is limited by the requirement of a rapid hydrogel gelation to achieve high-resolution printed patterns, resulting in low flow rates [125]. Recently, an optimization of the bioinks was operated to increase their adequate physicochemical and biocompatible properties before, during, and after 3D printing, which appeared essential to ensure the success of the 3D bioprinting [126]. In comparison to all these developments of 3D tissue structures from hydrogels, an in situ aligned/micro-topographical structure was recently produced using a decellularized extracellular matrix (dECM) as a biochemical component and a modified 3D cell printing process [127]. The dECM was derived from the decellularization of porcine skeletal muscles and chemically modified by methacrylate process to enhance mechanical stability. Following seeding in this hydrogel-free scaffold, C2C12 myoblasts were shown to be aligned and differentiated with a high degree of myotube formation. Another type of tissue-specific dECM bioinks was presented to efficiently induce cell differentiation and tissue development [128]. Indeed, compared to conventional collagen bioink-printed constructs, this new bioink contributed to significant improved cell proliferation, greater myogenic gene expression as well as higher mature myotube formation characterized by the presence of striated band pattern and contraction in response to electrical stimulation.

Overall, these findings demonstrated that the use of bioinks with 3D cell printing technology could provide a sufficient myogenic microenvironment and the appropriate architecture of native muscle tissue.

## Exploitation of engineered muscle tissues in the study of farm animal species and prospects of application

Ageing negatively affects muscle regeneration and muscle stem cell potential, resulting in muscle tissue pauperization or sarcopenia [129, 130]. Several studies have shown that aged SCs present functional alterations, among which capacity to activate, proliferate and differentiate [131, 132]. In Pig, Polyethylene glycol (PEG)-based hydrogel scaffold was used to complement data on the impact of aging on tissue regeneration efficiency [133]. For that purpose, adult skeletal muscle-derived pericytes, known to contribute to the regeneration process, were isolated from young (piglet) and adult (boar) pigs. Cell suspension was mixed with PEGylated fibrinogen (PF) precursor solution, added into cylindrical silicon molds and placed under a long-wave UV lamp to allow transition of PF into a gel [57]. PF has been described as having a remarkable influence on differentiation of myogenic progenitors by providing a 3D microenvironment suitable for muscle fiber development [57, 58]. Culture medium was added immediately to the polymerized hydrogels to ensure cell growth. The plugs were cultured for 24 h in serum-supplemented growth medium and then transferred into the serum-depleted differentiation medium for 5 days in order to promote muscle fiber formation. For in vivo experiments, molds were immediately implanted subcutaneously in the backs of mice. In vitro, pericytes from boars had similar morphology and colony forming capacity to piglet ones, but an impaired ability to form myotubes and capillary-like structures. Interestingly, the use of a PEG-based hydrogel scaffold to support adult pericytes was also shown to significantly improve their myogenic differentiation and angiogenic potential in vitro and in vivo, positioning it as a suitable niche to promote skeletal muscle regeneration and blood vessel growth.

**Figure 2:**
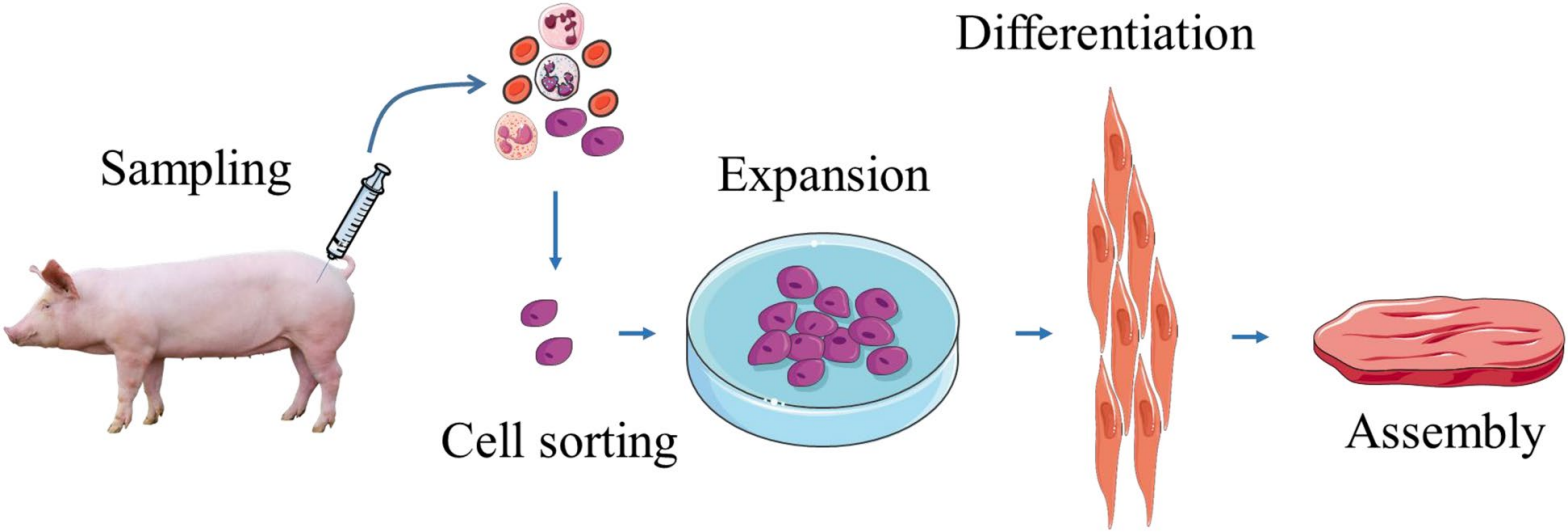
**Main steps required for production of cultured meat from an animal biopsy.** Satellite cells are isolated from animal muscle biopsy and subsequently in vitro expanded. When a sufficient quantity of myogenic cells is obtained, differentiation of myoblasts is induced. Resulting myotubes start producing proteins to form functional myocytes which can be then assembled with known food processing methods (mixing, molding) to form cultured meat.

**Figure 3:**
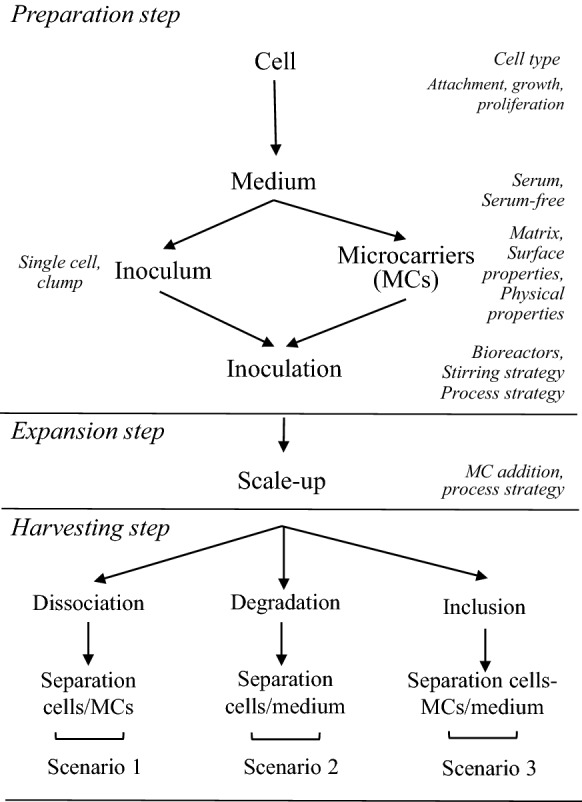
**Process requirements and variables for microcarriers based bioprocesses in three scenarios.** During the preparation and expansion steps, microcarriers (MCs) serve as a temporary substrate for cell attachment and proliferation before being separated from the cells following the scale up through dissociation (scenario 1) and degradation (scenario 2) or being embedded in the final product if they are edible (scenario 3) (adapted from [135]).

Several methods of producing cultured meat have been proposed and different cell types have been considered, including embryonic stem cells, induced pluripotent stem cells, mesenchymal stem cells and SCs [134]. In 3D cultured meat, the cells are grown on a scaffold, which is a component that directs its structure and order. The ideal scaffold is edible so the meat does not have to be removed, and periodically moves to stretch the developing muscle, thereby simulating the animal body during normal development. In addition, it must maintain flexibility to do not detach from the developing myotubes and favor vascularization to allow normal development of muscle tissue (Figure 2). Bovine muscle stem cells seem the most straightforward suitable candidates for this purpose. Potential attributes of microcarriers (MCs) to be used for meat production can be considered according to three different scenarios represented in Figure 3. In scenario 1, MCs are used as temporary substrates for the SC expansion and need to be removed at the end of the scale-up process. There are two important prerequisites in this case, namely the need of MCs to provide a high detachment yield and an easy separation from the cells. Concerning the scenario 2, MCs also serve as a temporary substrate for SC expansion but instead of being separated at the end of the scale-up process as done in the scenario 1 they can be degraded at a prior stage. In this case, the dissociation step can be replaced by a MC degradation step to obtain a single cell suspension. Finally, in the scenario 3, MCs are composed of edible materials and so can be embedded in the final product. As opposed to the previous scenario where MCs are considered as a food contact material, here they should comply with regulations for use as a food ingredient or additive. Indeed, besides supporting cell growth, an edible MC would also be part of the final product and might affect the sensory attributes of the meat product, such as taste, color or texture [135]. In order to more resemble the natural product, the Israeli company named MeaTech proposed to use a 3D printing techniques with the aim of improving the texture of cultured meat [136].

Overall, the use of 3D in vitro models of skeletal muscle tissue may be considered of highly interest in four areas of application corresponding to i) the in-depth characterization of myogenic progenitors in a large wide of species including Pig, Poultry and Fish to investigate the notion of tissue-resident cell diversity; ii) a better understanding of the deregulations affecting the myogenic progenitors in production species; iii) the study of the impact of vaccination on growth and inflammatory component in livestock animals; and iv) the provision of a study model for the meat maturation process.

## Abbreviations

2D: Two-dimensional
3D: Three-dimensional
Ach: Acetylcholine
AChR: Acetylcholine receptor
CD: Cluster of differentiation
dECM: Decellularized extracellular matrix
ECM: Extracellular matrix
GelMa: Methacrylated gelatin
MDC: Muscle-derived cell
MHC: Myosin heavy chain
MN: Motor neuron
NMJ: Neuromuscular junction
PDMS: Polydimethylsiloxane
PEG: Polyethylene glycol
PF: Pegylated fibrinogen
SC: Satellite cell
Sca-1: Stem cell antigen-1
UV: Ultraviolet

## References

[1] Janssen I, Heymsfield SB, Wang ZM, Ross R (2000). Skeletal muscle mass and distribution in 468 men and women aged 18–88 yr. J Appl Physiol.

[2] Wigmore PM, Evans DJ (2002). Molecular and cellular mechanisms involved in the generation of fiber diversity during myogenesis. Int Rev Cytol.

[3] Charge SB, Rudnicki MA (2004). Cellular and molecular regulation of muscle regeneration. Physiol Rev.

[4] Schmalbruch H, Lewis DM (2000). Dynamics of nuclei of muscle fibers and connective tissue cells in normal and denervated rat muscles. Muscle Nerve.

[5] Mauro A (1961). Satellite cell of skeletal muscle fibers. J Biophys Biochem Cytol.

[6] Seale P, Rudnicki MA (2000). A new look at the origin, function, and "stem-cell" status of muscle satellite cells. Dev Biol.

[7] Zammit PS, Partridge TA, Yablonka-Reuveni Z (2006). The skeletal muscle satellite cell: the stem cell that came in from the cold. J Histochem Cytochem.

[8] Grounds MD (1998). Age-associated changes in the response of skeletal muscle cells to exercise and regeneration. Ann N Y Acad Sci.

[9] Holterman CE, Rudnicki MA (2005). Molecular regulation of satellite cell function. Semin Cell Dev Biol.

[10] Katz B (1961). The terminations of the afferent nerve fibre in the muscle spindle of the frog. Philos Trans Royal Soc Lond [Biol].

[11] Laguens R (1963). Satellite cells of skeletal muscle fibers in human progressive muscular dystrophy. Virchows Arch Pathol Anat Physiol Klin Med.

[12] Shafiq SA, Gorycki MA, Milhorat AT (1967). An electron microscopic study of regeneration and satellite cells in human muscle. Neurology.

[13] Muir AR, Kanji AH, Allbrook D (1965). The structure of the satellite cells in skeletal muscle. J Anat.

[14] McLoon LK, Wirtschafter J (2003). Activated satellite cells in extraocular muscles of normal adult monkeys and humans. Invest Ophthalmol Vis Sci.

[15] Mesires NT, Doumit ME (2002). Satellite cell proliferation and differentiation during postnatal growth of porcine skeletal muscle. Am J Physiol Cell Physiol.

[16] Dodson MV, Martin EL, Brannon MA, Mathison BA, McFarland DC (1987). Optimization of bovine satellite cell-derived myotube formation in vitro. Tissue Cell.

[17] Gros J, Scaal M, Marcelle C (2004). A two-step mechanism for myotome formation in chick. Dev Cell.

[18] Halevy O, Piestun Y, Allouh MZ, Rosser BW, Rinkevich Y, Reshef R, Rozenboim I, Wleklinski-Lee M, Yablonka-Reuveni Z (2004). Pattern of Pax7 expression during myogenesis in the posthatch chicken establishes a model for satellite cell differentiation and renewal. Dev Dyn.

[19] Morrison JI, Loof S, He P, Simon A (2006). Salamander limb regeneration involves the activation of a multipotent skeletal muscle satellite cell population. J Cell Biol.

[20] Hammond CL, Hinits Y, Osborn DP, Minchin JE, Tettamanti G, Hughes SM (2007). Signals and myogenic regulatory factors restrict pax3 and pax7 expression to dermomyo-tome-like tissue in zebrafish. Dev Biol.

[21] Powell RL, Dodson MV, Cloud JG (1989). Cultivation and differentiation of satellite cells from skeletal muscle of the rainbow trout *Salmo gairdneri*. J Exp Zool.

[22] McCaig CD (1986). Myoblasts and myoblast-conditioned medium attract the earliest spinal neurites from frog embryos. J Physiol.

[23] Somers DG, Pearson ML, Ingles CJ (1975). Isolation and characterization of an alpha-amanitin-resistant rat myoblast mutant cell line possessing alpha-amanitin-resistant RNA polymerase II. J Biol Chem.

[24] Rando TA, Blau HM (1994). Primary mouse myoblast purification, characterization, and transplantation for cell-mediated gene therapy. J Cell Biol.

[25] Baroffio A, Hamann M, Bernheim L, Bochaton-Piallat ML, Gabbiani G, Bader CR (1996). Identification of self-renewing myoblasts in the progeny of single human muscle satellite cells. Differentiation.

[26] Yasin R, van Beers G, Bulien D, Thompson EJ (1976). A quantitative procedure for the dissociation of adult mammalian muscle into mononucleated cells. Exp Cell Res.

[27] Blanton JR, Grant AL, McFarland DC, Robinson JP, Bidwell CA (1999). Isolation of two populations of myoblasts from porcine skeletal muscle. Muscle Nerve.

[28] Buckingham ME, Cohen A, Gros F (1976). Cytoplasmic distribution of pulse-labelled poly(A)-containing RNA, particularly 26 S RNA, during myoblast growth and differentiation. J Mol Biol.

[29] Daubas P, Caput D, Buckingham M, Gros F (1981). A comparison between the synthesis of contractile proteins and the accumulation of their translatable mRNAs during calf myoblast differentiation. Dev Biol.

[30] Grounds MD, Yablonka-Reuveni Z, Partridge T (1993). Molecular and cellular biology of muscle regeneration. Molecular and Cell Biology of Muscular Dystrophy.

[31] Hay ED (1963). The fine structure of differentiating muscle in the salamander tail. Z Zellforsch Mikrosk Anat.

[32] Devoto SH, Melançon E, Eisen JS, Westerfield M (1996). Identification of separate slow and fast muscle precursor cells in vivo, prior to somite formation. Development.

[33] Rescan PY, Gauvry L, Paboeuf G (1995). A gene with homology to myogenin is expressed in developing myotomal musculature of the rainbow trout and in vitro during the conversion of myosatellite cells to myotubes. FEBS Lett.

[34] Eberli D, Soker S, Atala A, Yoo JJ (2009). Optimization of human skeletal muscle precursor cell culture and myofiber formation in vitro. Methods.

[35] Guo X, Greene K, Akanda N, Smith A, Stancescu M, Lambert S, Vandenburgh H, Hickman J (2014). In vitro differentiation of functional human skeletal myotubes in a defined system. Biomater Sci.

[36] Burkholder TJ, Fingado B, Baron S, Lieber RL (1994). Relationship between muscle fiber types and sizes and muscle architectural properties in the mouse hindlimb. J Morphol.

[37] Lieber RL, Friden J (2000). Functional and clinical significance of skeletal muscle architecture. Muscle Nerve.

[38] Cosgrove BD, Sacco A, Gilbert PM, Blau HM (2009). A home away from home: challenges and opportunities in engineering in vitro muscle satellite cell niches. Differentiation.

[39] Ghaemmaghami AM, Hancock MJ, Harrington H, Kaji H, Khademhosseini A (2012). Biomimetic tissues on a chip for drug discovery. Drug Discov Today.

[40] Oh CE, Antes K, Darby M, Song S, Starkschall G (1999). Comparison of 2D conventional, 3D conformal, and intensity-modulated treatment planning techniques for patients with prostate cancer with regard to target-dose homogeneity and dose to critical, uninvolved structures. Med Dosim.

[41] Dambach DM, Uppal H (2012). Improving risk assessment. Sci Transl Med.

[42] Bhatia SN, Ingber DE (2014). Microfluidic organs-on-chips. Nat Biotechnol.

[43] Ikemoto M, Fukada S, Uezumi A, Masuda S, Miyoshi H, Yamamoto H, Wada MR, Masubuchi N, Miyagoe-Suzuki Y, Takeda S (2007). Autologous transplantation of SM/C-2.6(+) satellite cells transduced with micro-dystrophin CS1 cDNA by lentiviral vector into mdx mice. Mol Ther.

[44] Obokata H, Kojima K, Westerman K, Yamato M, Okano T, Tsuneda S, Vacanti CA (2011). The potential of stem cells in adult tissues representative of the three germ layers. Tissue Eng Part A.

[45] Sarig R, Baruchi Z, Fuchs O, Nudel U, Yaffe D (2006). Regeneration and transdifferentiation potential of muscle-derived stem cells propagated as myospheres. Stem Cells.

[46] Arsic N, Mamaeva D, Lamb NJ, Fernandez A (2008). Muscle-derived stem cells isolated as non-adherent population give rise to cardiac, skeletal muscle and neural lineages. Exp Cell Res.

[47] Westerman KA, Penvose A, Yang Z, Allen PD, Vacanti CA (2010). Adult muscle 'stem' cells can be sustained in culture as free-floating myospheres. Exp Cell Res.

[48] Rouger K, Larcher T, Dubreil L, Deschamps JY, Le Guiner C, Jouvion G, Delorme B, Lieubeau B, Carlus M, Fornasari B, Theret M, Orlando P, Ledevin M, Zuber C, Leroux I, Deleau S, Guigand L, Testault I, Le Rumeur E, Fiszman M, Chérel Y (2011). Systemic delivery of allogenic muscle stem cells induces long-term muscle repair and clinical efficacy in duchenne muscular dystrophy dogs. Am J Pathol.

[49] Wei Y, Li Y, Chen C, Stoelzel K, Kaufmann AM, Albers AE (2011). Human skeletal muscle-derived stem cells retain stem cell properties after expansion in myosphere culture. Exp Cell Res.

[50] Penvose A, Westerman KA (2012). Sca-1 is involved in the adhesion of myosphere cells to alphaVbeta3 integrin. Biol Open.

[51] Westerman KA (2015). Myospheres are composed of two cell types: one that is myogenic and a second that is mesenchymal. PLoS One.

[52] van den Brink SC, Sage F, Vertesy A, Spanjaard B, Peterson-Maduro J, Baron CS, Robin C, van Oudenaarden A (2017). Single-cell sequencing reveals dissociation-induced gene expression in tissue subpopulations. Nat Methods.

[53] Liu JY, Swartz DD, Peng HF, Gugino SF, Russell JA, Andreadis ST (2007). Functional tissue-engineered blood vessels from bone marrow progenitor cells. Cardiovasc Res.

[54] Liu A, Chen S, Cai S, Dong L, Liu L, Yang Y, Guo F, Lu X, He H, Chen Q, Hu S, Qiu H (2014). Wnt5a through noncanonical Wnt/JNK or Wnt/PKC signaling contributes to the differentiation of mesenchymal stem cells into type II alveolar epithelial cells in vitro. PLoS One.

[55] Vandenburgh HH, Karlisch P, Farr L (1988). Maintenance of highly contractile tissue-cultured avian skeletal myotubes in collagen gel. Vitro Cell Dev Biol.

[56] Dennis RG, Kosnik PE (2000). Excitability and isometric contractile properties of mammalian skeletal muscle constructs engineered in vitro. Vitro Cell Dev Biol Anim.

[57] Fuoco C, Salvatori ML, Biondo A, Shapira-Schweitzer K, Santoleri S, Antonini S, Bernardini S, Tedesco FS, Cannata S, Seliktar D, Cossu G, Gargioli C (2012). Injectable polyethylene glycol-fibrinogen hydrogel adjuvant improves survival and differentiation of transplanted mesoangioblasts in acute and chronic skeletal-muscle degeneration. Skelet Muscle.

[58] Rizzi R, Bearzi C, Mauretti A, Bernardini S, Cannata S, Gargioli C (2012). Tissue engineering for skeletal muscle regeneration. Muscles Ligaments Tendons J.

[59] Bach AD, Beier JP, Stern-Staeter J, Horch RE (2004). Skeletal muscle tissue engineering. J Cell Mol Med.

[60] Rosso F, Giordano A, Barbarisi M, Barbarisi A (2004). From cell-ECM interactions to tissue engineering. J Cell Physiol.

[61] De Coppi P, Bellini S, Conconi MT, Sabatti M, Simonato E, Gamba PG, Nussdorfer GG, Parnigotto PP (2006). Myoblast-acellular skeletal muscle matrix constructs guarantee a long-term repair of experimental full-thickness abdominal wall defects. Tissue Eng.

[62] Turner NJ, Yates AJ, Weber DJ, Qureshi IR, Stolz DB, Gilbert TW, Badylak SF (2010). Xenogeneic extracellular matrix as an inductive scaffold for regeneration of a functioning musculotendinous junction. Tissue Eng Part A.

[63] Juhas M, Ye J, Bursac N (2016). Design, evaluation, and application of engineered skeletal muscle. Methods.

[64] Hong Y, Takanari K, Amoroso NJ, Hashizume R, Brennan-Pierce EP, Freund JM, Badylak SF, Wagner WR (2012). An elastomeric patch electrospun from a blended solution of dermal extracellular matrix and biodegradable polyurethane for rat abdominal wall repair. Tissue Eng Part C Methods.

[65] Wolf MT, Dearth CL, Sonnenberg SB, Loboa EG, Badylak SF (2015). Naturally derived and synthetic scaffolds for skeletal muscle reconstruction. Adv Drug Deliv Rev.

[66] Velleman SG (1999). The role of the extracellular matrix in skeletal muscle development. Poult Sci.

[67] Osses N, Brandan E (2002). ECM is required for skeletal muscle differentiation independently of muscle regulatory factor expression. Am J Physiol Cell Physiol.

[68] Okano T, Matsuda T (1997). Hybrid muscular tissues: preparation of skeletal muscle cell-incorporated collagen gels. Cell Transplant.

[69] Bian W, Bursac N (2009). Engineered skeletal muscle tissue networks with controllable architecture. Biomaterials.

[70] Huang YC, Dennis RG, Larkin L (1985). Baar K (2005) Rapid formation of functional muscle in vitro using fibrin gels. J Appl Physiol.

[71] Yamamoto Y, Ito A, Fujita H, Nagamori E, Kawabe Y, Kamihira M (2011). Functional evaluation of artificial skeletal muscle tissue constructs fabricated by a magnetic force-based tissue engineering technique. Tissue Eng Part A.

[72] Shadrin IY, Khodabukus A, Bursac N (2016). Striated muscle function, regeneration, and repair. Cell Mol Life Sci.

[73] Hinds S, Bian W, Dennis RG, Bursac N (2011). The role of extracellular matrix composition in structure and function of bioengineered skeletal muscle. Biomaterials.

[74] Rhim C, Lowell DA, Reedy MC, Slentz DH, Zhang SJ, Kraus WE, Truskey GA (2007). Morphology and ultrastructure of differentiating three-dimensional mammalian skeletal muscle in a collagen gel. Muscle Nerve.

[75] Grassl ED, Oegema TR, Tranquillo RT (2002). Fibrin as an alternative biopolymer to type-I collagen for the fabrication of a media equivalent. J Biomed Mater Res.

[76] Ross JJ, Tranquillo RT (2003). ECM gene expression correlates with in vitro tissue growth and development in fibrin gel remodeled by neonatal smooth muscle cells. Matrix Biol.

[77] Collet JP, Shuman H, Ledger RE, Lee S, Weisel JW (2005). The elasticity of an individual fibrin fiber in a clot. Proc Natl Acad Sci U S A.

[78] Yang L, van der Werf KO, Koopman BF, Subramaniam V, Bennink ML, Dijkstra PJ, Feijen J (2007). Micromechanical bending of single collagen fibrils using atomic force microscopy. J Biomed Mater Res A.

[79] Dietrich F, Lelkes PI (2006). Fine-tuning of a three-dimensional microcarrier-based angiogenesis assay for the analysis of endothelial-mesenchymal cell co-cultures in fibrin and collagen gels. Angiogenesis.

[80] Maffioletti SM, Sarcar S, Henderson ABH, Mannhardt I, Pinton L, Moyle LA, Steele-Stallard H, Cappellari O, Wells KE, Ferrari G, Mitchell JS, Tyzack GE, Kotiadis VN, Khedr M, Ragazzi M, Wang W, Duchen MR, Patani R, Zammit PS, Wells DJ, Eschenhagen T, Tedesco FS (2018). Three-dimensional human iPSC-derived artificial skeletal muscles model muscular dystrophies and enable multilineage tissue engineering. Cell Rep.

[81] Juhas M, Bursac N (2014). Roles of adherent myogenic cells and dynamic culture in engineered muscle function and maintenance of satellite cells. Biomaterials.

[82] Afshar Bakooshli M, Lippmann ES, Mulcahy B, Iyer N, Nguyen CT, Tung K, Stewart BA, van den Dorpel H, Fuehrmann T, Shoichet M, Bigot A, Pegoraro E, Ahn H, Ginsberg H, Zhen M, Ashton RS, Gilbert PM (2019). A 3D culture model of innervated human skeletal muscle enables studies of the adult neuromuscular junction. Elife.

[83] Hansen A, Eder A, Bonstrup M, Flato M, Mewe M, Schaaf S, Aksehirlioglu B, Schwoerer AP, Uebeler J, Eschenhagen T (2010). Development of a drug screening platform based on engineered heart tissue. Circ Res.

[84] Rao L, Qian Y, Khodabukus A, Ribar T, Bursac N (2018). Engineering human pluripotent stem cells into a functional skeletal muscle tissue. Nat Commun.

[85] Khodabukus A, Baar K (2009). Regulating fibrinolysis to engineer skeletal muscle from the C2C12 cell line. Tissue Eng Part C Methods.

[86] Vandenburgh HH (1988). A computerized mechanical cell stimulator for tissue culture: effects on skeletal muscle organogenesis. Vitro Cell Dev Biol.

[87] Hosseini V, Ahadian S, Ostrovidov S, Camci-Unal G, Chen S, Kaji H, Ramalingam M, Khademhosseini A (2012). Engineered contractile skeletal muscle tissue on a microgrooved methacrylated gelatin substrate. Tissue Eng Part A.

[88] Ostrovidov S, Jiang J, Sakai Y, Fujii T (2004). Membrane-based PDMS microbioreactor for perfused 3D primary rat hepatocyte cultures. Biomed Microdevices.

[89] Ostrovidov S, Sakai Y, Fujii T (2011). Integration of a pump and an electrical sensor into a membrane-based PDMS microbioreactor for cell culture and drug testing. Biomed Microdevices.

[90] Madden L, Juhas M, Kraus WE, Truskey GA, Bursac N (2015). Bioengineered human myobundles mimic clinical responses of skeletal muscle to drugs. Elife.

[91] Roche SM, Gumucio JP, Brooks SV, Mendias CL, Claflin DR (2015). Measurement of maximum isometric force generated by permeabilized skeletal muscle fibers. J Vis Exp.

[92] Pedrotty DM, Koh J, Davis BH, Taylor DA, Wolf P, Niklason LE (2005). Engineering skeletal myoblasts: roles of three-dimensional culture and electrical stimulation. Am J Physiol Heart Circ Physiol.

[93] Flaibani M, Boldrin L, Cimetta E, Piccoli M, De Coppi P, Elvassore N (2009). Muscle differentiation and myotubes alignment is influenced by micropatterned surfaces and exogenous electrical stimulation. Tissue Eng Part A.

[94] Huang YC, Dennis RG, Baar K (2006). Cultured slow vs. fast skeletal muscle cells differ in physiology and responsiveness to stimulation. Am J Physiol Cell Physiol.

[95] Donnelly K, Khodabukus A, Philp A, Deldicque L, Dennis RG, Baar K (2010). A novel bioreactor for stimulating skeletal muscle in vitro. Tissue Eng Part C Methods.

[96] Khodabukus A, Prabhu N, Wang J, Bursac N (2018). In vitro tissue-engineered skeletal muscle models for studying muscle physiology and disease. Adv Healthc Mater.

[97] Powell CA, Smiley BL, Mills J, Vandenburgh HH (2002). Mechanical stimulation improves tissue-engineered human skeletal muscle. Am J Physiol Cell Physiol.

[98] Handschin C, Mortezavi A, Plock J, Eberli D (2015). External physical and biochemical stimulation to enhance skeletal muscle bioengineering. Adv Drug Deliv Rev.

[99] Ostrovidov S, Ahadian S, Ramon-Azcon J, Hosseini V, Fujie T, Parthiban SP, Shiku H, Matsue T, Kaji H, Ramalingam M, Bae H, Khademhosseini A (2017). Three-dimensional co-culture of C2C12/PC12 cells improves skeletal muscle tissue formation and function. J Tissue Eng Regen Med.

[100] Koffler J, Kaufman-Francis K, Shandalov Y, Egozi D, Pavlov DA, Landesberg A, Levenberg S (2011). Improved vascular organization enhances functional integration of engineered skeletal muscle grafts. Proc Natl Acad Sci U S A.

[101] Sasagawa T, Shimizu T, Sekiya S, Haraguchi Y, Yamato M, Sawa Y, Okano T (2010). Design of prevascularized three-dimensional cell-dense tissues using a cell sheet stacking manipulation technology. Biomaterials.

[102] Derby B (2012). Printing and prototyping of tissues and scaffolds. Science.

[103] Murphy SV, Atala A (2014). 3D bioprinting of tissues and organs. Nat Biotechnol.

[104] Moroni L, Burdick JA, Highley C, Lee SJ, Morimoto Y, Takeuchi S, Yoo JJ (2018). Biofabrication strategies for 3D in vitro models and regenerative medicine. Nat Rev Mater.

[105] Zorlutuna P, Annabi N, Camci-Unal G, Nikkhah M, Cha JM, Nichol JW, Manbachi A, Bae H, Chen S, Khademhosseini A (2012). Microfabricated biomaterials for engineering 3D tissues. Adv Mater.

[106] Garg K, Bowlin GL (2011). Electrospinning jets and nanofibrous structures. Biomicrofluidics.

[107] Gilbert-Honick J, Ginn B, Zhang Y, Salehi S, Wagner KR, Mao HQ, Grayson WL (2018). Adipose-derived stem/stromal cells on electrospun fibrin microfiber bundles enable moderate muscle reconstruction in a volumetric muscle loss model. Cell Transplant.

[108] Soliman E, Bianchi F, Sleigh JN, George JH, Cader MZ, Cui Z, Ye H (2018). Engineered method for directional growth of muscle sheets on electrospun fibers. J Biomed Mater Res A.

[109] Wang L, Wu Y, Guo B, Ma PX (2015). Nanofiber yarn/hydrogel core-shell scaffolds mimicking native skeletal muscle tissue for guiding 3D myoblast alignment, elongation, and differentiation. ACS Nano.

[110] Weinandy S, Laffar S, Unger RE, Flanagan TC, Loesel R, Kirkpatrick CJ, van Zandvoort M, Hermanns-Sachweh B, Dreier A, Klee D, Jockenhoevel S (2014). Biofunctionalized microfiber-assisted formation of intrinsic three-dimensional capillary-like structures. Tissue Eng Part A.

[111] Patil P, Szymanski JM, Feinberg AW (2016). Defined micropatterning of ECM protein adhesive sites on alginate microfibers for engineering highly anisotropic muscle cell bundles. Adv Mater Technol.

[112] Nakanishi J, Takarada T, Yamaguchi K, Maeda M (2008). Recent advances in cell micropatterning techniques for bioanalytical and biomedical sciences. Anal Sci.

[113] Guijt RM, Breadmore MC (2008). Maskless photolithography using UV LEDs. Lab Chip.

[114] Li B, He M, Ramirez L, George J, Wang J (2016). Multifunctional hydrogel microparticles by polymer-assisted photolithography. ACS Appl Mater Interfaces.

[115] Norris SCP, Tseng P, Kasko AM (2016). Direct gradient photolithography of photodegradable hydrogels with patterned stiffness control with submicrometer resolution. ACS Biomater Sci Eng.

[116] Albisetti E, Carroll KM, Lu X, Curtis JE, Petti D, Bertacco R, Riedo E (2016). Thermochemical scanning probe lithography of protein gradients at the nanoscale. Nanotechnology.

[117] Lindquist NC, Nagpal P, McPeak KM, Norris DJ, Oh SH (2012). Engineering metallic nanostructures for plasmonics and nanophotonics. Rep Prog Phys.

[118] Xia Y, Whitesides GM (1998). Soft Lithography. Angew Chem Int Ed Engl.

[119] Kang MS, Lee SH, Park WJ, Lee JE, Kim B, Han DW (2020). Advanced techniques for skeletal muscle tissue engineering and regeneration. Bioengineering (Basel).

[120] Derakhshanfar S, Mbeleck R, Xu K, Zhang X, Zhong W, Xing M (2018). 3D bioprinting for biomedical devices and tissue engineering: A review of recent trends and advances. Bioact Mater.

[121] Agarwala S (2016). A perspective on 3D bioprinting technology: present and future. Am J Eng Appl Sci.

[122] Costantini M, Testa S, Mozetic P, Barbetta A, Fuoco C, Fornetti E, Tamiro F, Bernardini S, Jaroszewicz J, Święszkowski W, Trombetta M, Castagnoli L, Seliktar D, Garstecki P, Cesareni G, Cannata S, Rainer A, Gargioli C (2017). Microfluidic-enhanced 3D bioprinting of aligned myoblast-laden hydrogels leads to functionally organized myofibers in vitro and in vivo. Biomaterials.

[123] Jang J, Park HJ, Kim SW, Kim H, Park JY, Na SJ, Kim HJ, Park MN, Choi SH, Park SH, Kwon SM, Kim PJ, Cho DW (2017). 3D printed complex tissue construct using stem cell-laden decellularized extracellular matrix bioinks for cardiac repair. Biomaterials.

[124] Helps T, Taghavi M, Rossiter J (2019). Thermoplastic electroactive gels for 3D-printable artificial muscles. Smart Mater Struct.

[125] Merceron TK, Burt M, Seol YJ, Kang HW, Lee SJ, Yoo JJ, Atala A (2015). A 3D bioprinted complex structure for engineering the muscle-tendon unit. Biofabrication.

[126] Mredha MTI, Guo YZ, Nonoyama T, Nakajima T, Kurokawa T, Gong JP (2018). A facile method to fabricate anisotropic hydrogels with perfectly aligned hierarchical fibrous structures. Adv Mater.

[127] Kim W, Lee H, Lee J, Atala A, Yoo JJ, Lee SJ, Kim GH (2020). Efficient myotube formation in 3D bioprinted tissue construct by biochemical and topographical cues. Biomaterials.

[128] Choi YJ, Kim TG, Jeong J, Yi HG, Park JW, Hwang W, Cho DW (2016). 3D cell printing of functional skeletal muscle constructs using skeletal muscle-derived bioink. Adv Healthc Mater.

[129] Carosio S, Berardinelli MG, Aucello M, Musaro A (2011). Impact of ageing on muscle cell regeneration. Ageing Res Rev.

[130] García-Prat L, Sousa-Victor P, Muñoz-Cánoves P (2013). Functional dysregulation of stem cells during aging: a focus on skeletal muscle stem cells. FEBS J.

[131] Conboy IM, Conboy MJ, Smythe GM, Rando TA (2003). Notch-mediated restoration of regenerative potential to aged muscle. Science.

[132] Baj A, Bettaccini AA, Casalone R, Sala A, Cherubino P, Toniolo AQ (2005). Culture of skeletal myoblasts from human donors aged over 40 years: dynamics of cell growth and expression of differentiation markers. J Transl Med.

[133] Fuoco C, Sangalli E, Vono R, Testa S, Sacchetti B, Latronico MV, Bernardini S, Madeddu P, Cesareni G, Seliktar D, Rizzi R, Bearzi C, Cannata SM, Spinetti G, Gargioli C (2014). 3D hydrogel environment rejuvenates aged pericytes for skeletal muscle tissue engineering. Front Physiol.

[134] Post MJ (2012). Cultured meat from stem cells: challenges and prospects. Meat Sci.

[135] Bodiou V, Moutsatsou P, Post MJ (2020). Microcarriers for upscaling cultured meat production. Front Nutr.

[136] Meatech. https://meatech3d.com/.

